# Biological Markers of Auditory Gap Detection in Young, Middle-Aged, and Older Adults

**DOI:** 10.1371/journal.pone.0010101

**Published:** 2010-04-09

**Authors:** Bernhard Ross, Bruce Schneider, Joel S. Snyder, Claude Alain

**Affiliations:** 1 Rotman Research Institute, Baycrest Centre, Toronto, Ontario, Canada; 2 Department of Medical Biophysics, University of Toronto, Toronto, Ontario, Canada; 3 Department of Psychology, University of Toronto, Toronto, Ontario, Canada; 4 Department of Psychology, University of Nevada, Las Vegas, Nevada, United States of America; University of Washington, United States of America

## Abstract

The capability of processing rapid fluctuations in the temporal envelope of sound declines with age and this contributes to older adults' difficulties in understanding speech. Although, changes in central auditory processing during aging have been proposed as cause for communication deficits, an open question remains which stage of processing is mostly affected by age related changes. We investigated auditory temporal resolution in young, middle-aged, and older listeners with neuromagnetic evoked responses to gap stimuli with different leading marker and gap durations. Signal components specific for processing the physical details of sound stimuli as well as the auditory objects as a whole were derived from the evoked activity and served as biological markers for temporal processing at different cortical levels. Early oscillatory 40-Hz responses were elicited by the onsets of leading and lagging markers and indicated central registration of the gap with similar amplitude in all three age groups. High-gamma responses were predominantly related to the duration of no-gap stimuli or to the duration of gaps when present, and decreased in amplitude and phase locking with increasing age. Correspondingly, low-frequency activity around 200 ms and later was reduced in middle aged and older participants. High-gamma band, and long-latency low-frequency responses were interpreted as reflecting higher order processes related to the grouping of sound items into auditory objects and updating of memory for these objects. The observed effects indicate that age-related changes in auditory acuity have more to do with higher-order brain functions than previously thought.

## Introduction

Spoken words are complex acoustic signals characterized by rapid fluctuations in the speech envelope with periods of high sound energy interspersed with low-energy periods (gaps). In particular, variations in the gap duration (e.g., the voice onset time) for consonant-vowel combinations convey important information, which determine whether people hear, for example, the phoneme \ta\ or the phoneme \da\. A two-alternative forced-choice procedure in which listeners chose between two stimuli (one containing a gap) is commonly used in laboratory tests of the listeners' ability to detect a short interruption in an otherwise continuous sound. In such experiment the gap is bounded by a leading and a lagging marker. The gap stimuli used in these studies are thought to be acoustically analogous to speech events like voice-onset time for consonants and may index one of the temporal processing mechanisms involved in speech perception [1].

An established model for the limits (i.e., thresholds) in gap detection posits that the sensation of the leading marker preceding the gap does not vanish immediately after it ends but rather decays with a certain time constant and interferes with the subsequent sensation related to the onset of the lagging marker [2]. For pure tone or noise markers of same type, previous studies have indicated that listeners can discriminate between two stimuli (i.e., gap and no-gap) differing by only 2–4 ms in the temporal envelope [3]–[7]. Such small limits for gap detection have been explained in terms of the properties of the auditory periphery [4]. Additionally, the sound onset elicits a strong sensation, which interacts with detecting a gap shortly after sound onset and gap detection thresholds are elevated for short leading marker durations [8]. Such a model based on auditory time constants limiting the registration of leading and lagging markers has received support from animal studies showing that neurons in the inferior colliculus responded preferentially to the onsets of the markers [9], [10]. This suggests that registration of marker onsets is a key acoustical feature for gap detection. Multi-unit responses to the onset of the lagging marker recorded from the cat auditory cortex closely resembled behavioural threshold [11] suggesting that cortical function may be the limiting factor for gap detection performance. Moreover, such data from cat auditory cortex demonstrated that gap-detection thresholds increase the closer the gap is to sound onset.

Behavioral studies indicate that older adults require greater separation between leading and trailing markers in order to detect a gap [12]–[14] or to report hearing two separate sounds [15], [16]. Interestingly, the extent of age-related differences in gap detection threshold appears to be linked to the duration of the leading marker. As the separation between the onset of the sound and the onset of the gap increases, age-differences in gap thresholds decrease. Indeed, older listeners are able to detect gaps as short as young listeners when the separation between the onset of the sound and the onset of the gap is sufficiently large [14], [17].

One possible mechanism that could account for the pattern of age-related decrements in gap detection would be a prolongation of time constants of the auditory system. An age-related increase in the time constant of the response to the leading marker would increase the degree to which a response to the initial marker masks the response to the lagging marker [17]. One implicit assumption of such a model, that has not been tested, concerns the processing and representation of leading and lagging markers at various levels of auditory cortices. If age-related increases in gap detection are due to increases in the time-constants of sub-cortical processes, then these age-differences should be reflected equally in neural activity at all cortical stages. Alternatively, aging may affect auditory cortical processing differently. The current study was designed to specifically test this hypothesis by measuring neuromagnetic brain activity using magnetoencephalography (MEG).

Prior research using MEG has revealed the transient middle-latency evoked field (i.e. gamma response) to the onset of the lagging marker of a gap stimulus, which was derived as the difference wave between the response to gap stimulus and continuous sound of same duration [18]. The transient responses in that study increased with increasing gap duration, and with the duration of the leading marker, and were correlated with behavioral measures of gap detection. Because of its short refractory time, the middle-latency component of the auditory evoked field (AEF) has been shown to be useful in elucidating the neural mechanisms associated with auditory processing of fine temporal structure [19]. Using pairs of brief tone pips the investigators in that study showed that a later wave emerged and increased in amplitude with increasing temporal and spectral separation of the tone pips. This additional wave could indicate the perception of two sound objects instead of a single item. Consistent with this interpretation, Joliot et al. [20] showed that auditory evoked 40-Hz oscillations were modified when a pair of click stimuli was separated by more than 13 ms, which was close to the threshold for perceptual separation of the clicks. These studies indicate that fast transient changes in neuroelectric activity are important biological markers for the processing of the fine temporal structure in sound. The terms ‘neuromagnetic middle-latency response’ and ‘40-Hz (gamma) oscillations’ were used interchangeably in those studies for what were likely closely related phenomena [21].

However, is it reasonable to conclude that these auditory evoked responses mediate perception of a gap? An alternative hypothesis for decline in temporal acuity during aging would be that the sound features are registered successfully: however, the ability to access the acoustical information required for the formation of a meaningful auditory object might be impaired. Recent findings suggest that neural encoding of acoustical primitives such as the duration of sound [22] or harmonic relationships [23] are only marginally impaired by age. The goals of our current study were to identify biological markers of temporal processing in central hearing and to compare those across the adult lifespan. We analyzed multiple, functionally different components of the AEF separately to explore which stages of information processing are affected by aging. If aging primarily impairs auditory object formation, we would expect that the representation of information at early stages of cortical processing would be relatively unaffected by age, and that age related differences would be manifested in neural activity indicating higher order auditory processes associated with object formation. We studied neuromagnetic auditory evoked responses in young, middle-aged and older adults and identified oscillatory gamma-band activity with sources in bilateral auditory cortices that was specific to the onset of the lagging marker in the gap stimulus. The size of the response and its complexity, as expressed by the number of oscillatory cycles, increased with longer duration of the gap and with longer duration of the leading marker. This stimulus-response relation was consistent across age groups. However, an oscillatory response at higher gamma frequency and long latency responses, both hypothesized to be related to higher order auditory processing, declined across age groups. The results provide evidence that normal aging affects auditory processing on a higher level than previously thought.

## Results

### Neuromagnetic source analysis

**Figure 1:**
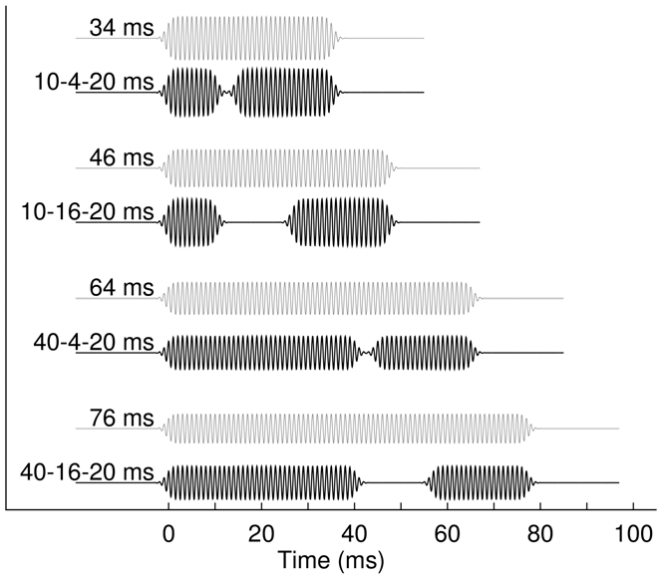
Waveforms of the auditory stimuli. The sounds of 34, 46, 64, and 76 ms total duration, respectively, were either continuous tones or contained a temporal gap. The gap lengths were 4 or 16 ms, the length of the leading marker 10 or 40 ms, and the trailing marker was always 20 ms long. The stimulus amplitudes were adjusted for equal energy.

Neuromagnetic responses were recorded while the participants listened to sequences of tonal stimuli, half of which contained a temporal gap (Figure 1). The sources of brain activity were localized based on the grand-averaged AEF lumped over stimulus types. The locations of single equivalent dipoles could be successfully estimated for all individual subjects for the oscillatory 40-Hz response and the N1_m_ wave of the AEF. Source coordinates were consistent across the age groups; and distances between the mean source locations for the different age groups in any direction were less than 6 mm and no distance was significantly different from zero. Sources in the right hemisphere were located more anteriorly than in the left hemisphere for both the 40-Hz response (4.5 mm, t(41) = 4.9, p<0.0001) and the N1_m_ response (5.1 mm, t(41) = 4.0, p = 0.0003). Such anatomical asymmetry between left and right auditory cortex is a general finding across subjects [24], [25] and the statistical significance for this distance on the order of 5.0 mm indicates highly consistent dipole localization results. Sources of the 40-Hz response were located more superiorly than the N1_m_ sources in right (6.0 mm, t(41) = 3.88, p = 0.0004) and left hemisphere (6.1 mm, t(41) = 3.36, p = 0.0017). The absolute source coordinates in the Talairach coordinate system were (−44, −13, 12 mm) and (51, −17, 11 mm) for the right and left N1_m_ sources and (−45, −14, 12 mm) and (52, −18, 16 mm) for the right and left 40-Hz sources.

### Multiple types of evoked responses

**Figure 2:**
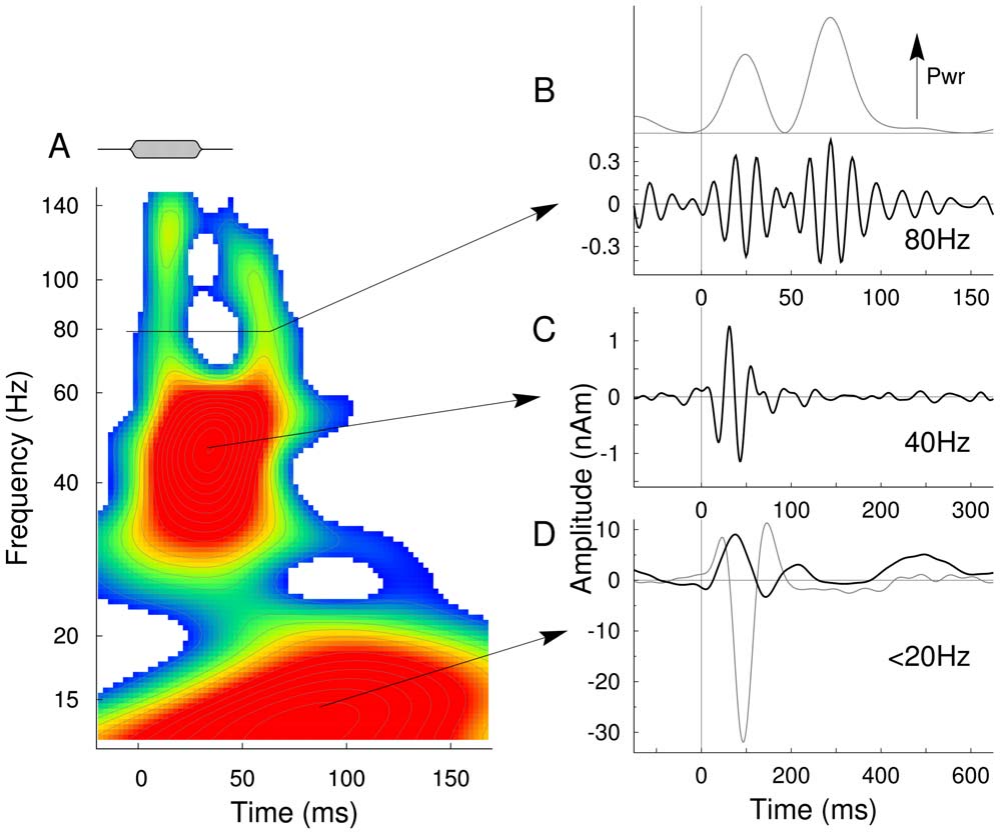
Overview of the types of auditory evoked responses. **A**: The time-frequency map of inter-trial coherence in the responses to the 34-ms no-gap stimulus shows three main effects: the low frequency response below 20 Hz, the transient gamma band response at 40 Hz, and high-gamma responses at 80 Hz and above. **B**: The time series of activity in the high-gamma band shows two bursts of oscillations, which are expressed in two peaks in the time series of the signal power (upper trace). **C**: The transient 40-Hz response is likely equivalent to the middle-latency response. **D**: The low frequency response (black) shows predominantly a positive peak resembling a P1m response. The N1_m_ -P2_m_ response is strongly reduced because of fast stimulus repetition. For comparison the response to same stimuli at longer SOA (1500ms) is shown as gray line.

The temporal dynamics of the spectral components of cortical source activity were analyzed using a wavelet transform. The representative time-frequency map of a response to the 34-ms no-gap stimulus in Figure 2 illustrates three different types of evoked responses, which can be separated in the frequency domain using appropriate filters. The dominant spectral peak below 20 Hz between 50 and 100 ms in time is mainly expressed as a P1_m_ response as shown in Figure 2D. Because the inter-stimulus interval between 120 and 320 ms was much shorter than the refractory period of the N1_m_ and P2_m_ wave [26], both were almost absent in the current data. For comparison, the P1_m_–N1_m_–P2_m_ as observed with the same stimulus but longer SOA of 1500 ms [22] is overlaid on the low-frequency waveform in Figure 2D. The dominant peak in the spectrogram above 40 Hz with latency below 50 ms corresponds to the transient gamma-band response shown after band-pass filtering between 18 and 80 Hz in Figure 2C. Above 80 Hz the spectrogram shows two peaks separated in time, one occurring after stimulus onset and the second after stimulus offset. The time course of the 80-Hz high-pass filtered response shows two distinct short bursts of oscillation, which can be seen in the time course of the signal power in Figure 2B. Based on the observation of time-frequency maps for all age groups as shown in Figure 2 we selected the frequency band of 72–98 Hz as representative for the high-gamma band for further analysis.

### Difference waves

**Figure 3:**
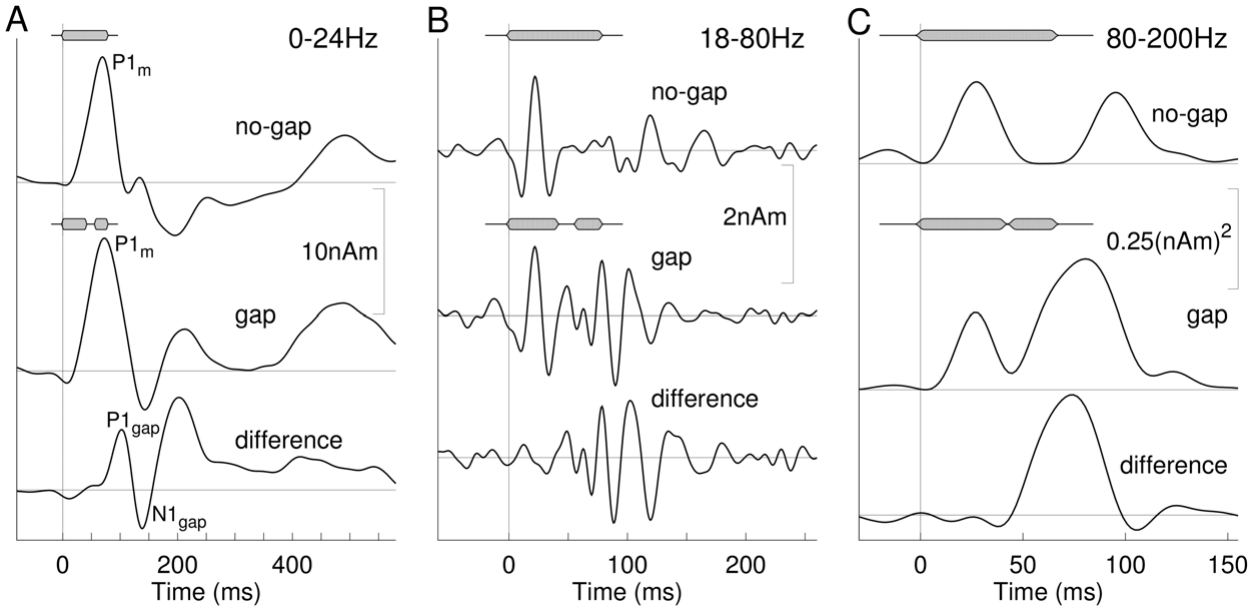
Comparison of responses to no-gap and gap stimuli using difference waveforms. As an example, the responses to the stimulus with long leading marker and long gap (40-16-20 ms) are shown as grand average for the young group. **A**: Waveforms of the 0–24 Hz low-pass filtered responses show similar P1_m_ waves for the gap and no-gap condition. In the difference waveform (lower trace), a clear P1_m_ -N1_m_ -P2_m_ like pattern appears with delayed latency compared to the onset response. **B**: Waveforms of the 18–80 Hz band-pass filtered responses. The transient onset response is almost identical for the gap and no-gap response and is cancelled out in the difference waveform, which shows a more complex pattern of oscillation than the onset response. **C**: Signal power of the 80–200 Hz filtered high-gamma band data. Two bursts of high-gamma activity occur for the gap and no-gap stimulus, respectively. The second gamma burst for the gap stimulus is largest and appears earlier than the second burst for the no-gap stimulus. The difference in signal power shows predominantly a single burst of high-gamma activity.

To isolate waveforms of specific responses to a gap in the sound we subtracted the response to a no-gap stimulus from the responses to the corresponding gap stimulus (i.e., gap minus no-gap). Such subtractions are illustrated in Figure 3 for responses obtained in the young group for the gap stimulus with long leading marker and long gap duration (40-16-20 ms) and the no-gap stimulus of 76 ms duration. The 24-Hz low-pass filtered response showed predominantly a P1_m_ wave for both the gap and no-gap stimulus. Those initial responses were cancelled out in the difference wave, which showed a P1_m_–N1_m_–P2_m_ like wave with delayed latency compared to the stimulus onset responses (Figure 3A). The onset portion of the 18-80-Hz filtered response was almost identical for the gap and no-gap responses and was cancelled out in the difference wave (Figure 3B). Similarly, the signal power in the high-gamma band, above 80 Hz, showed two bursts of oscillation for both stimulus types with the second burst for the gap stimulus occurring earlier than the second burst for the no-gap stimulus. The difference waveform was dominated by a single burst of high-gamma oscillations. The high-gamma bursts were clearly separated in time, even for the shortest sound duration, thus the peak amplitudes and latencies of the original signal were analyzed and the difference operation was not necessary (Figure 3C).

### *Long latency evoked responses*

The predominant component in the long-latency responses was the P1_m_ wave, which did not differ in amplitude across stimulus types (F<1). However, the ANOVA revealed a main effect of age (F(2,38) = 5.4, p = 0.008) with larger amplitudes in the middle-aged compared to young (p = 0.025) and in older compared to younger participants (p = 0.0014), whereas the amplitude difference between older and middle-aged adults did not reach significance. The P1_m_ latency increased with age (F(2,38) = 4.3, p = 0.02) from 45 ms in the young, to 58 ms in the older group (p = 0.004), whereas the latency of 55 ms in the middle-aged group was not significantly different from the other groups.

**Figure 4:**
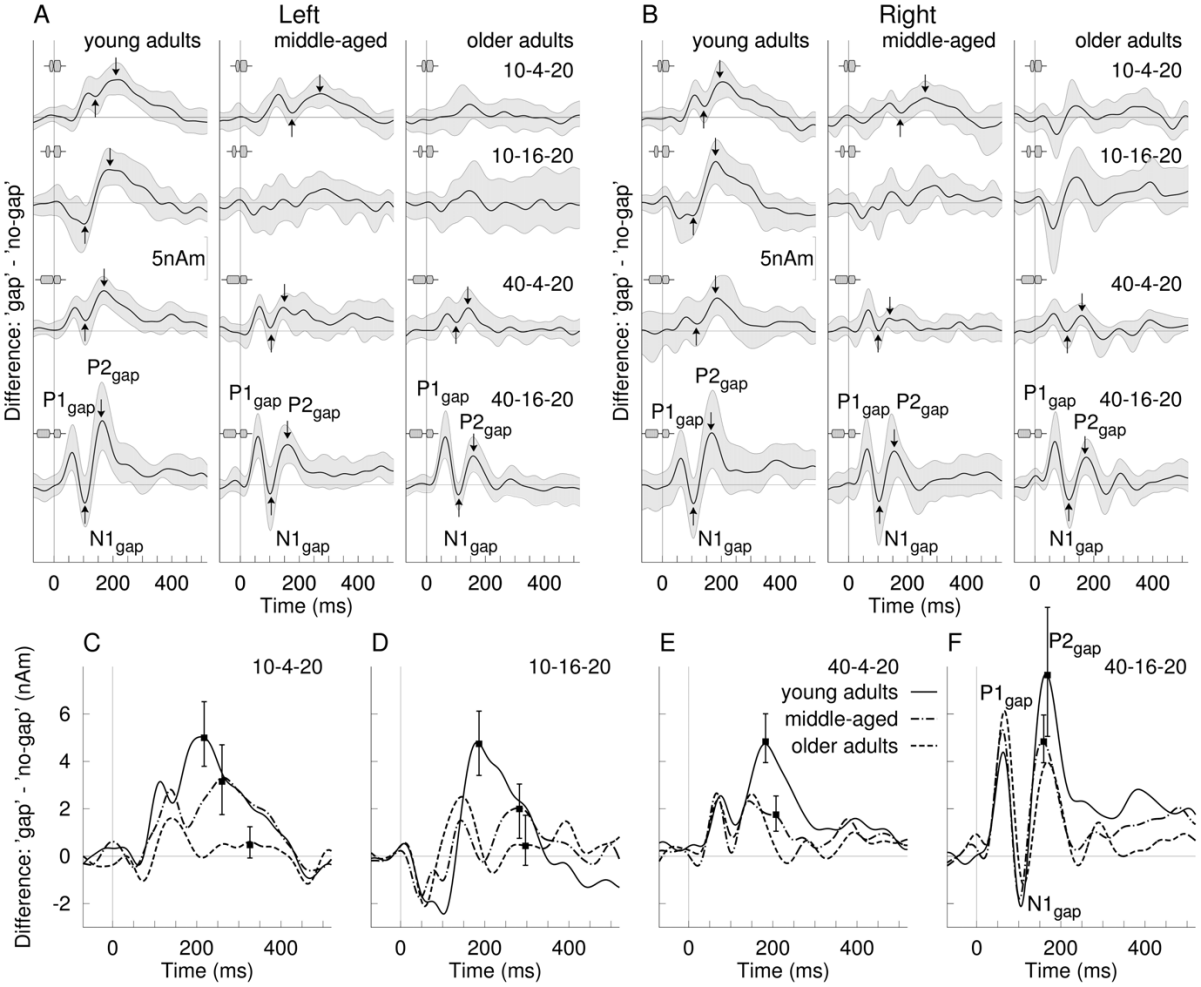
Difference waves between 24-Hz low-pass filtered responses to gap and no-gap stimuli. **A**: Comparison of difference waves observed in the left hemisphere between the three age groups. The thick lines represent the group mean difference waves and the gray shaded areas above and below indicate the 95% confidence interval for the group mean. The time scale was adjusted that zero corresponds to the onset of the trailing marker. P1_m_ -N1_m_ -P2_m_ waves can be observed for all age groups in the responses to the 40-16-20 ms stimulus. In general the response size declined with shorter gap duration and shorter duration of the leading marker. Upward arrows point to possible N1_gap_ waves and downward arrows P2_gap_ waves. **B**: Difference waves in the right hemisphere resembled the left hemispheric responses in wave configuration, amplitudes and latencies. **C–F**: Overlay of grand mean difference waves across hemispheres for the three age groups and the four stimulus conditions.

The waveforms of group mean differences between 24-Hz low-pass filtered responses to gap and no-gap stimuli are shown in Figure 4A for the left and in Figure 4B for the right hemisphere. For the stimulus with long leading marker and long gap duration (40-16-20 ms) the difference waveforms show a three-phasic complex resembling a P1_m_–N1_m_–P2_m_ response, which have been labeled P1_gap_–N1_gap_–P2_gap_. The gray shaded areas above and below the waveforms indicate the 95% confidence ranges for the group mean. The confidence limits show that the amplitudes of the P1_gap_ and P2_gap_ were significantly different from zero, except for the right hemispheric response in the young group, and all N1_gap_ peaks were significantly more negative than the corresponding P1_gap_ and P2_gap_ peaks. The origin of the time axis was adjusted to the onset of the trailing markers of the stimulus. According to this time scale, the group mean P1_gap_, N1_gap_, and P2_gap_ latencies and the 95% confidence limits for the mean were 61±4, 105±5, and 168±8 ms, respectively in the young group, 60±4, 104±4, and 163±8 in the middle-aged, and 64±4, 113±5, and 172±6 ms in the older group. The peak latencies were not significantly different between the age groups.

The group mean difference waves averaged across hemispheres are illustrated in Figure 4C–F. The most prominent effect of age was for the stimulus with long leading marker and long gap duration (40-16-20 ms), which became obvious in the latency range around 200 ms (Figure 4F). The P2_gap_ amplitude decreased with increasing age (F(2,38) = 4.93, p = 0.012) whereas the peak latency (178 ms) did not differ across the age groups. In contrast, the P1_gap_ amplitudes increased with age, as has been observed for the P1_m_ onset response; however, the increase did not reach significance. The shorter gap duration stimulus (40-4-20 ms) resulted in strong decrease in the response amplitude for all age groups. Again, the waveforms around 200 ms showed the largest age effect. The response in the young group at 190 ms latency was larger than in middle-aged and older participants (F(2,38) = 13.0, p<.0001). Similarly, the peak amplitude of the response to the stimulus with short leading marker and long gap duration (10-16-20 ms) was largest in the younger group (F(2,38) = 12.0, p<.0001). For the short gap duration (10-4-20 ms) the main effect of age (F(2,38) = 9.5, p = 0.0005) revealed larger responses in the young than middle-aged participants (p<.02) and larger responses in middle-aged than older adults (p<0.001). In summary, the effect of age on the long latency response was mainly expressed as decreased amplitudes in the latency range of the P2_gap_ response for increasing age.

### Transient 40-Hz responses

**Figure 5:**
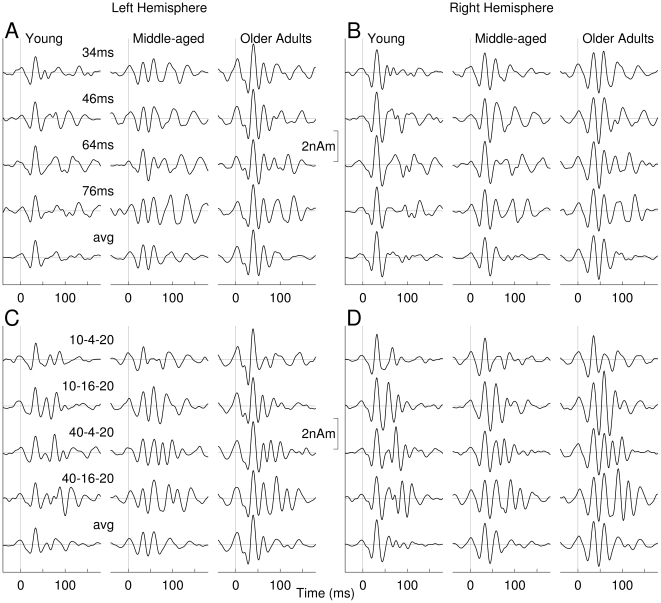
Waveforms of transient gamma band responses for the three age groups, eight stimulus types and left and right hemispheres. (Time zero refers to the stimulus onset.) **A**: Responses to no-gap stimuli as observed in the left hemisphere. The bottom trace shows the response waveform averaged across the four stimuli with different duration. **B**: Right hemispheric responses to no-gap stimuli. **C–D**: Responses to gap-stimuli accordingly.

Figure 5 shows the grand-averaged source waveforms of the transient 40-Hz response for the left and right hemisphere in young, middle-aged and older adults for the no-gap (Figure 5A–B) and gap stimuli (Figure 5C–D). Common to gap and no-gap responses, all age groups, and left and right hemispheres, was that the early parts of the waveforms (<50 ms) were almost identical regardless the stimulus type. The early response, representing the onset of the stimulus was followed by a pattern of positive and negative waves of variable complexity and latency according to the stimulus type. The later parts of the responses represent the offset response in case of a no-gap stimulus and a combination of responses to the gap and offset responses in case of the gap stimulus. Averaging across the responses to the four no-gap stimuli of different duration cancels the later parts of the response because of different latencies. The resulting waveforms, shown in the bottom traces in Figure 5 represent mainly the onset portion of the response. Similarly, averaging across the responses to the four gap stimuli primarily canceled out the gap responses. The remaining onset responses were almost identical for gap and no-gap stimuli. In contrast, the patterns of waveforms were noticeably different between age groups and between hemispheres. A characteristic feature seems to be that the complexity of waveforms (i.e. the number of positive and negative peaks) increased with increasing age.

### Gap difference waves

**Figure 6:**
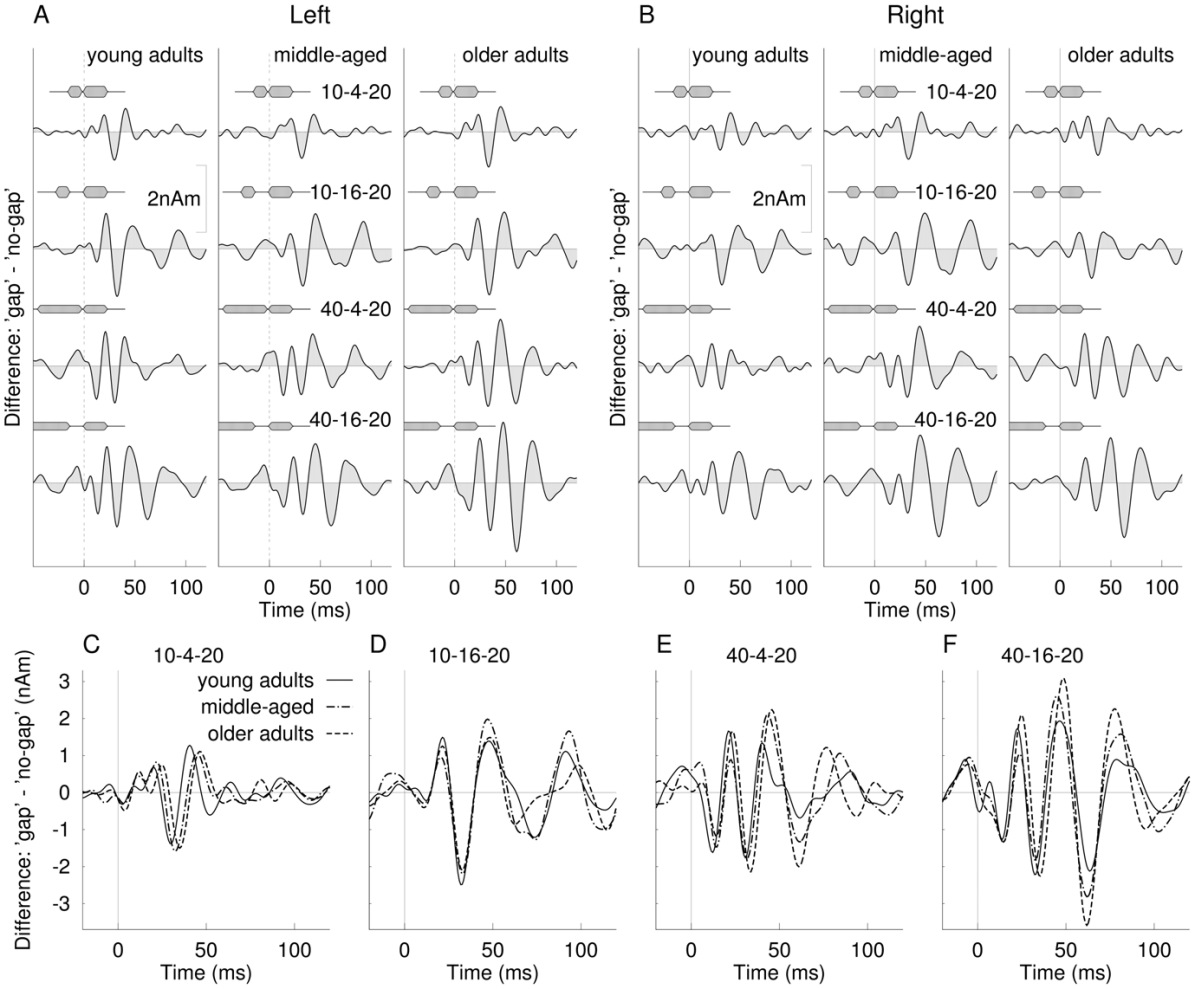
Difference waves between gap and no-gap stimuli for the transient 40-Hz response, the three age groups, and four different stimuli. Time zero of all time scales is adjusted to the onset of the trailing marker. **A**: Difference waves observed in the left and **B**: in the right hemisphere. **C–F**: Overlay of difference waves observed in the three age groups averaged across left and right hemispheres.

The similarity between the onset responses to gap and no-gap stimuli supports the concept of calculating difference waveforms for extracting the effect of a gap because equal onset responses for both types of stimuli will cancel each other out in the differences. The resulting difference waveforms are shown in Figure 6. The time scales were adjusted so time zero corresponds to the onset of the lagging marker. The difference waves for all stimulus types, age groups and both hemispheres showed a pattern of positive and negative deflections in the interval after onset of the lagging marker compared to the immediately preceding time interval indicating responses to the leading marker. The response patterns increased in complexity for increasing gap duration and increasing duration of the leading marker. Also the response complexity increases with increasing age, although the changes across age groups seem to be small relative to differences across stimulus types. To compare between age groups the difference waves were averaged across hemispheres and overlaid for the different stimulus types in Figure 6C–F. Almost identical responses waveforms, except small latency differences, were observed for the three age groups especially for the initial part of the response (<50 ms), whereas the responses noticeably changed between stimuli of different durations for the leading marker and the gap.

### Effect of aging on the 40-Hz response

**Figure 7:**
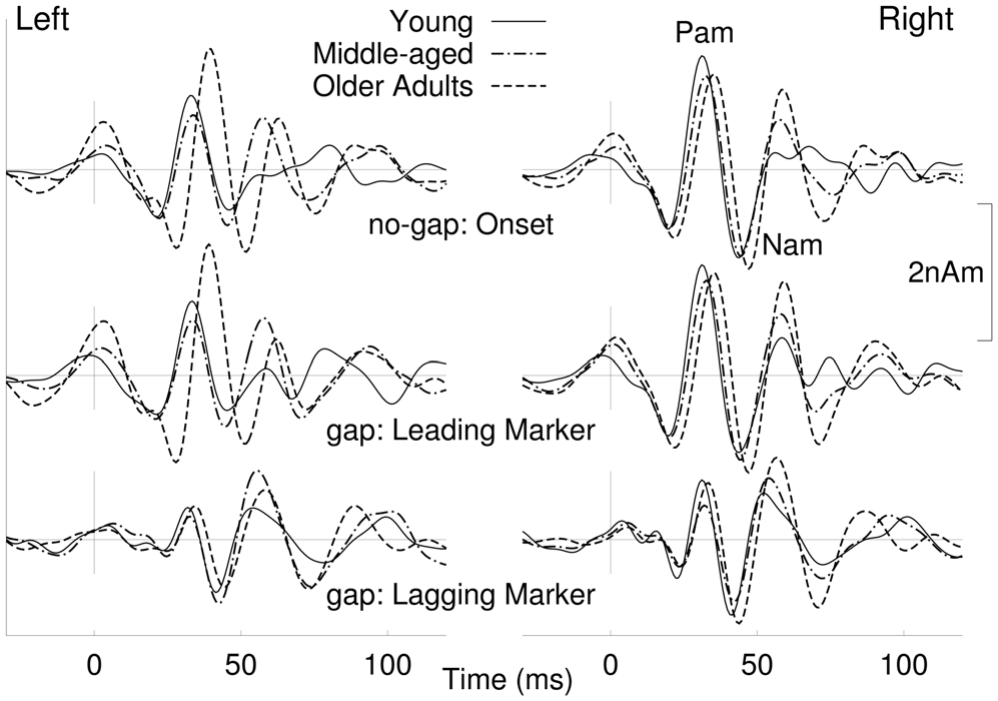
Overlay of grand averaged responses to the onset of no-gap stimuli for the three age groups (top traces). The most prominent positive and negative going waves are labeled in the right hemispheric responses as Pa_m_ and Na_m_ according to the nomenclature used for middle latency responses. Grand averaged responses to the onset of the leading markers of gap stimuli are shown in the middle traces and responses to the lagging markers obtained from the difference waves in the bottom traces.

For visualizing the main effects of age, the 40-Hz responses to the no-gap stimulus were averaged regardless of the stimulus duration and overlaid for the three age groups in the upper traces of Figure 7. Accordingly, the responses to the four gap stimuli were averaged and the difference waveforms with latencies adjusted to the onset of the lagging marker as shown in the middle and lower traces in Figure 7. Especially in the right hemisphere the response pattern was consistent for the onset of no-gap stimuli, the onset of the leading marker in the gap stimuli, and the onset of the lagging marker in the gap stimuli. The left hemispheric responses for the onset of no-gap stimuli and onsets of the leading marker in gap stimuli were very similar, but different in the wave pattern and latencies from the onset response to the lagging marker. However, responses to the onset of the lagging marker resembled each other across hemispheres.

The early cycle of the 40-Hz response comprises positive and negative waves labeled as Pa_m_ and Na_m_ (Figure 7). Both peaks could be identified in the individual waveforms in all participants except in one older adult. ANOVA applied to amplitude and latencies of both peaks revealed a main effect of age on the Na_m_ peak latency (F(2,38) = 8.78, p = .0007), and the Pa_m_ peak latency (F(2,38) = 22.2, p<.0001) with older adults showing delayed latencies compared to young adults. An interaction between age and hemisphere (Na_m_: F(2,38) = 4.09, p = .024, Pa_m_: F(2,38) = 4.68, p = .015) reflected greater age differences in the left than in the right hemisphere. There was also a main effect of age for the Na_m_ amplitude (F(2,38) = 6.98, p = .0026) and Pa_m_ amplitude (F(2,38) = 5.14, p = .011) with older adults showing larger amplitude compared to young or middle-aged adults. An age x hemisphere interaction (Na_m_: F(2,38) = 3.89, p = .029; Pa_m_: F(2,38) = 7.96, p = .0013) revealed that age-related amplitude differences occurred in the left but not the right hemisphere. The waveforms shown in Figure 7 indicate that the age effect was mainly expressed as larger and delayed response in the left hemisphere in the group of older adults compared to both other groups.

Besides changes in amplitudes and latencies of the initial part of the 40-Hz response the morphology of the oscillatory pattern changed across age group in the later part of the response. The right hemispheric response in the young group can be described by 1.5 cycles of oscillation, whereas the middle-aged participants showed 2 cycles and the older group 2.5 cycles of oscillation. This tendency toward a more complex oscillatory 40-Hz response with increasing age was consistently seen after onset of the no-gap and gap stimulus and after onset of the lagging marker in the gap stimulus.

### High-gamma band responses to no-gap stimuli

Responses were more consistent in the right than left hemisphere. This was expressed in larger values of the phase statistics in the right hemisphere for all stimulus types in all age groups (t(23) = 4.74, p<0.0001). Thus, the responses obtained in the right hemisphere only will be reported here.

**Figure 8:**
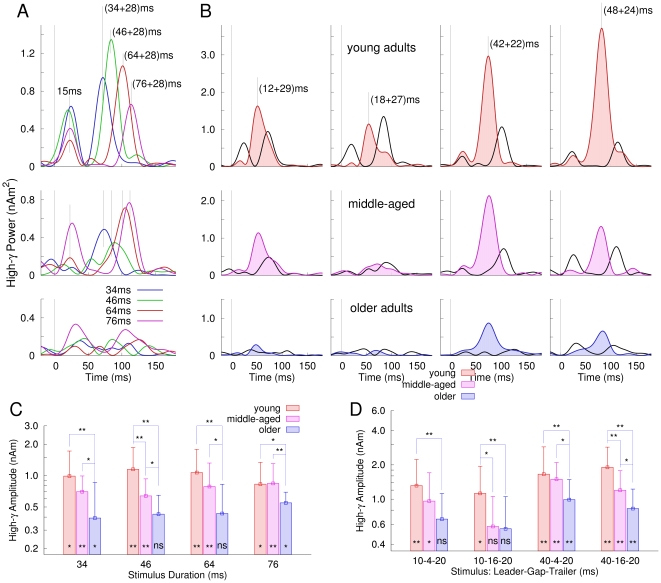
Time courses of activity in the higher gamma band (72–98 Hz) in the right auditory cortex. **A**: The responses to the no-gap stimuli exhibit two peaks, the first 15 ms after stimulus onset, the second 28 ms after stimulus offset. The response amplitudes decline with increasing age. **B**: Time courses of high-gamma responses to gap stimuli are shown in different color for the age groups in comparison to responses to the no-gap stimuli shown as black lines. The main peak in the responses to gap stimuli appears at latency between those of the onset and offset response in the corresponding no-gap stimuli. Responses to gap-stimuli were in general larger than responses to no-gap stimuli (note the different scales on the y-axes). **C**: Bar graphs representing the peak amplitude of the offset response to no-gap stimuli for the three age groups. The error bars denote the upper 95% confidence limits for the group mean. Results of the analysis of phase coherence across each group are indicated at the base of each bar (*: .05≤p<0.01, **: p≤0.01, ns: not significant). **D**: Bar graphs of the amplitudes of high-gamma responses to gap stimuli.

**Figure 9:**
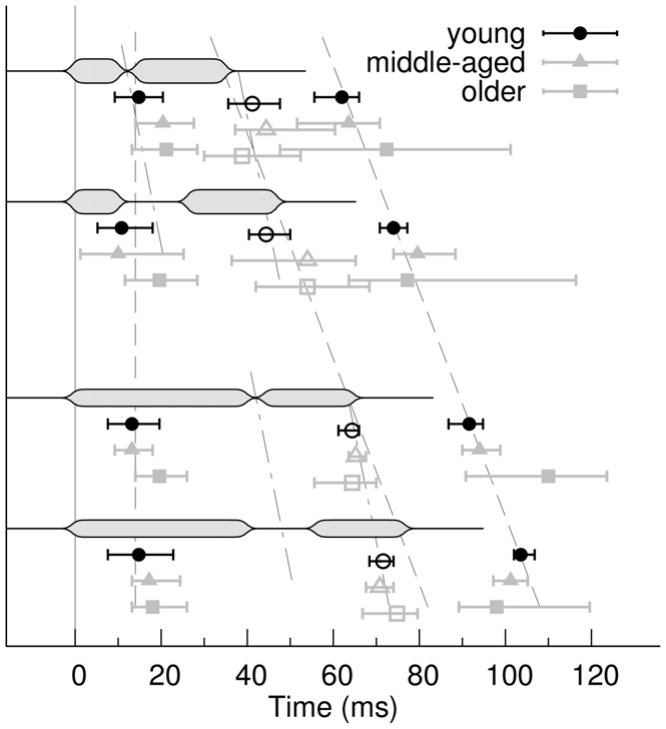
Group mean latencies of the high-gamma peak. Gap responses are marked with open symbols, onset and offset responses to the no-gap stimuli with filled symbols. The error bars denote the 95% confidence intervals for the group means. The dashed lines are parallels to the time points of sound onset and sound offset indicating that the latencies of the response to no-gap stimuli are well explained by a constant delay added to onset or offset latency. The dash-dotted lines are parallels to the mid points of the gaps and suggest that the latency of the gap responses (open symbols) are better explained by a constant delay with respect to the gap mid point.

The time courses of neural activity at high-gamma frequency, band-pass filtered between 72 and 98 Hz, showed two peaks for the no-gap stimuli. The first peak occurred at latency of 15 ms with respect to the sound onset for all stimuli and the second peak occurred with progressively prolonged latency for stimuli with increased duration (Figure 8A). Regression analysis, applied to the peak latency data in the younger group, showed that the measured peak latencies were well explained (R = 0.985) by the model that the latency of the second peak equals the sound duration plus a constant delay. The mean time delay with respect to the stimulus offset was 27.6 ms (Figure 9). Thus, the two bursts of oscillations occurred with constant latencies with respect to the stimulus onset and offset, respectively. The amplitudes of the offset responses were larger than the onset responses (p<0.008 for all stimulus durations), which was different from the 40-Hz response. The latter showed strongly reduced offset compared to onset components (Figure 5A–B). The following data analysis was focused on the offset component of the high-gamma response.

The amplitude of the offset response, a phase statistic indicating consistency within the group, and results of t-tests comparing the amplitudes between age groups are reported in Table 1 and visualized with bar graphs in Figure 8C. For all stimulus durations the size of the offset response was significantly larger in the younger group compared to older adults as well larger in middle-aged compared to the older group. Furthermore, the phase statistics testing consistency of the response within groups were significant for the young and middle-aged groups for all stimulus durations; however, it failed for the three shortest durations in the older group. Both measures indicated a decline in the high-gamma responses with increasing age.

**Table 1:** Amplitudes of the high-gamma following the offset of the no-gap stimuli or the gap in the gap stimuli for the three age groups (y: young, ma: middle-aged, o: older), phase coherence nR^2^ across the groups and p-value for the phase statistics, and results of t-tests for between group comparisons.

Stimulus	Group	Amplitude	NR^2^	P	Between group comparisons	
34ms	y	1.02 nAm	3.58	0.026	y>ma:	t(20) = 1.49, n.s.
	ma	0.72 nAm	4.58	0.0088	y>o:	t(24) = 2.99, p = 0.0031
	o	0.36 nAm	3.38	0.032	ma>o:	t(21) = 2.51, p = 0.010
46ms	y	1.18 nAm	7.14	0.0005	y>ma:	t(21) = 2.81, p = 0.0053
	ma	0.62 nAm	5.49	0.0032	y>o:	t(19) = 4.13, p = 0.0003
	o	0.43 nAm	2.13	0.12 (ns)	ma>o:	t(20) = 2.18, p = 0.021
64ms	y	1.10 nAm	6.15	0.0016	y>ma:	t(26) = 1.36, n.s.
	ma	0.87 nAm	4.74	0.0073	y>o:	t(23) = 3.34, p = 0.0014
	o	0.43 nAm	1.00	0.37 (ns)	ma>o:	t(20) = 2.24, p = 0.018
76ms	y	0.85 nAm	4.15	0.014	y>ma:	t(26) = −0.21, n.s.
	ma	0.88 nAm	7.12	0.0005	y>o:	t(18) = 2.23, p = 0.020
	o	0.55 nAm	4.51	0.01	ma>o:	t(13) = 2.67, p = 0.010
10_4_20ms	y	1.40 nAm	4.86	0.0067	y>ma:	t(26) = 1.23, n.s.
	ma	1.12 nAm	4.22	0.013	y>o:	t(22) = 2.78, p = 0.0054
	o	0.65 nAm	2.11	0.12 (ns)	ma>o:	t(18) = 1.45, n.s.
10_16_20ms	y	1.16 nAm	3.22	0.038	y>ma:	t(26) = 2.36, p = 0.013
	Ma	0.56 nAm	1.12	0.33 (ns)	y>o:	t(25) = 2.92, p = 0.0036
	O	0.52 nAm	2.13	0.07 (ns)	ma>o:	t(23) = 0.75, n.s.
40_4_20ms	Y	1.69 nAm	10.3	<0.0001	y>ma:	t(25) = 0.74, n.s.
	Ma	1.49 nAm	8.43	0.0001	y>o:	t(20) = 2.67, p = 0.0071
	O	0.97 nAm	6.55	0.001	ma>o:	t(19) = 2.51, p = 0.011
40_16_20ms	Y	1.96 nAm	15.2	<0.0001	y>ma:	t(25) = 2.86, p = 0.0041
	Ma	1.19 nAm	6.74	0.0007	y>o:	t(22) = 4.69, p<0.0001
	O	0.85 nAm	4.92	0.006	ma>o:	t(20) = 2.02, p = 0.028

### High gamma-band responses to gap stimuli

The high-gamma responses to the gap stimuli showed a dominant peak with latencies between the latencies of the onset and offset responses for the no-gap stimulus (Figure 8B). The maximum of high-gamma activity occurred around the offset of the stimulus but earlier than the maximum in the offset responses to the no-gap stimulus (Figure 9). The peak latencies were delayed by 28 ms with respect to the center of the gap for the stimuli with short leading marker and 23 ms for the long leading marker (Figure 8B). The widely overlapping error bars for the group mean peak latencies in Figure 9 indicate that the peak latencies were not significantly different between age groups.

Statistics for the peak amplitudes are reported in Table 1 and visualized with bar graphs in Figure 8D. For all stimulus types the size of the high-gamma response to the gap was significantly larger in younger compared to older adults. For the long duration gaps the responses in young adults were larger than in middle-aged and for the long leading marker duration stimulus the middle-aged showed larger responses than older adults. Furthermore, the phase statistics, testing consistency of the response within the group, was significant for the young group for all stimulus duration, however failed for the two short gap stimuli in the older group and for the long gap with short leading marker in the middle-aged group. Both measures indicated a decline in the high-gamma responses with increasing age as was found for offset responses.

## Discussion

### Transient gamma-band response

We will discuss our results with respect to the model that proposes that the early transient 40-Hz response results from recurrent oscillations in a thalamocortical network [27], [28]. Experimental evidence for this model has been derived from MEG analysis demonstrating cortical sources of the 40-Hz response [29] and contributions from subcortical areas with a time shift of 2–3 ms consistent with thalamocortical conduction delay [30]. The thalamocortical subsystem is specifically tuned to the 40-Hz range because of cortical interneurons and thalamic neurons involved in the network show intrinsic oscillations at this frequency [31], [32]. The thalamocortical network for generation of gamma oscillations is well documented and consists in principle of two recurrent loops between thalamus and cortex [28], [33], [34]. In the first loop, inputs from specific thalamic nuclei projects to layer-IV cortical interneurons and returns back to the thalamus. Recurrent activations along this loop result in a pattern of 40-Hz oscillations. A parallel oscillatory network is formed by a loop consisting of non-specific thalamic nuclei, which project to cortical layer I and receives recurrent cortico-thalamic projections. Synchronization between specific and non-specific recurrent loops provides a mechanism of binding multiple auditory events into an auditory object for further processing [28]. With MEG we most likely record the effect of intra cellular current flow of post-synaptic activity in apical dendrites of pyramidal cells involved in both thalamocortical loops. However, given the temporal order of activation, we assume that the early part of the transient 40-Hz oscillations observed in our study is generated in the specific thalamocortical loop.

Peak latencies and amplitudes of the early part of the 40-Hz oscillations were consistent across the stimulus types and were similar for the onset of the leading and the lagging marker. This means that the first part of the transient 40-Hz oscillations signals the onset of an auditory event at the level of thalamocortical processing. The later part of the oscillations is likely related to binding auditory items into an auditory object.

In terms of detecting a gap in sound, our data showed that the onset of the leading marker elicited about one and a half cycles of oscillation and this resembled almost perfectly the responses to the onset of the no-gap stimulus. For the gap stimulus the onset of the lagging marker elicited a similar pattern of oscillation with peak latency and amplitude comparable to the onset response. Whereas the responses to the leading marker or the no-gap stimulus were of equal amplitudes for all different durations (Figure 5), the size of the response to the lagging marker increased with increasing duration of the gap and leading marker. This can be explained with refractoriness within the same neuronal population involved in signaling the onsets of leading and lagging markers. With increasing duration of leading marker and gap there is more time for recovery before the response to the lagging marker is elicited. In summary, we conclude that the early part of the 40-Hz activity, as observed in the onset and difference waveforms, is related to signaling the onset of leading and lagging marker in a specific thalamocortical loop.

Most importantly, the 40-Hz responses to the leading marker were consistent across ages except a small increase in latency with increasing age. The onsets of the lagging markers were represented with almost identical amplitudes in all age groups. This was even the case for the short 10-4-20 ms stimulus, which was most difficult to detect. Thus we showed a biological marker indicating that the temporal acoustical features constituting the gap stimulus were represented at thalamocortical level even in the oldest participants and for the most difficult to detect gaps.

The later part of the transient 40-Hz component (>50 ms) showed a more complex behaviour. In general we observed two effects, which lead to increased complexity of the waveform, and both effects likely resulted from different underlying mechanisms. First, with increasing duration of the leading marker and the gap duration (i.e. increasing capability in detecting a temporal gap in the stimulus) the response waveform became more complex. Second, the later part of the transient 40-Hz response increased in complexity with age for both the onset of the leading and lagging markers. We could speculate that the emergence of additional cycles of oscillation as the gap stimulus became more detectable may result from additional oscillations in the non-specific thalamocortical loop related to the emergence of a second auditory object. Similarly, Joliot et al. [20] interpreted their finding of an additional cycle in the 40-Hz response to a click pair as indicating that the listeners perceived two sounds rather than independent responses to the two physical stimuli.

The effect of aging on the transient 40-Hz responses has been investigated mainly in terms of the auditory middle-latency response, and amplitudes and latencies of the Pa-Na-Pb waves have been studied. The common findings of increased latencies and increased amplitudes for Pa and Na waves [35], [36] with age have been reproduced in our study. The age-related increase in latency may reflect slowing in the conduction time within the ascending auditory pathways, which would be consistent with the general slowing hypothesis to account for age-related decline in perceptual and cognitive functions [37]. However, when comparing response amplitudes across age groups we need to consider the elevation in thresholds and loss of dynamic range that occurs with aging. These factors were unlikely to contribute significantly to the results presented here because the audiometric thresholds of older adults were elevated by no more than 15 dB relative to the younger participants and our stimuli were presented at fixed sound pressure level of 80 dB. Thus, it is unlikely that the tones were louder in older than middle-aged and younger participants. Diverse explanations exist, why the response size changes with aging; such as, an age-related loss in GABAergic inhibition would result in larger response amplitudes, and changes in volume conduction would affect the extra-cranial recording differently across age groups.

Moreover, the transient 40-Hz response became more complex (consisted of a larger number of cycles) as the stimulus became more discriminable, and as the age of the participants increased. This effect was evident for both the sound onset response and for the derived response to the onset of the lagging marker. Such effects have not been previously reported in studies of the effects of aging on the middle latency responses. The failure to notice these age-related changes might be due to experimenters focusing on a defined sequence of peaks or because a high rate of stimulus presentation obscured changes in the later part of the responses. An explanation on systems level could be that the continuing oscillations may result from less efficient coupling between the two thalamocortical loops. Loose coupling between the two loops may reduce damping to the specific loop and consequently prolonged oscillation. Age-related changes in neurotransmitter configurations might result in less damping in the thalamocortical network.

### High-gamma oscillations

The neural networks underlying oscillations at higher gamma frequencies became a focus of interest recently. At the level of auditory cortex, human electrocorticographic (ECoG) studies showed that a sharp increase of high-gamma oscillation 30–40 ms after sound onset, similar to the current data, was associated with higher cognitive function such as selective attention [38], memory-based matching [39], and discrimination [40]. Further intracranial recordings, showing phase coupling between low frequency theta and high frequency gamma oscillations, support the hypothesis that high frequency oscillations play a key role in communication across wide range cortical networks [41]. With the no-gap stimuli we found that the amplitudes of the offset responses were noticeably larger than the onset responses. This pattern differs from the pattern found for evoked responses at lower frequencies, which clearly indicate reduced offset compared to onset responses. The larger high-gamma offset response cannot be explained entirely as response to a physical change in the stimulus and suggest an endogenous contribution to the high-gamma response.

Whereas the peak latencies of the high-gamma offset responses to no-gap stimuli could be modeled with a constant delay added to the sound offset, a systematic relation of the peak latencies of the gap responses to a specific physical change in the stimulus was not as systematic. The peak latencies could be modeled at best with a constant delay after the mid point of the gap. Similar to the offset response to the no-gap stimuli, the amplitudes of the high-gamma responses were noticeably larger than responses to stimulus onset or offset. Again this is different from the early part of the 40-Hz response, which had been identified as signaling a physical change in the stimulus. Taken together latency and amplitude characteristics of the high-gamma gap responses suggest an endogenous nature of the response. Although further studies are necessary to explain the functional meaning of the high-gamma response we hypothesize that it is involved in evaluating the stimulus duration rather than signaling the sound offset, and/or in processing the gap rather than responding to a an abrupt change in the energy in the acoustical signal.

The high-gamma sound offset and gap responses decreased with increasing age, and the decrease was already significant between the middle-aged and younger group for the gap stimulus with longest gap duration. Given that the 40-Hz responses, which presumably encodes the elementary features of the gap, was not affected by aging, the decline in the high-gamma response region suggests that higher order processing of the temporal sound features does change with age.

The analysis of the high-gamma responses, like that of the 40-Hz responses, was entirely based on time and phase locked properties of the response signal with respect to the stimulus. Consistent latencies and phases of oscillatory activity across participants were assumed for the group analysis. An explanation for decreasing amplitudes of the high-gamma response with age could be that the response latencies are more variable between repeated trials in the older adults. Indeed, we found deteriorating phase consistency with increasing age. The interpretation that phase locking to the stimulus at higher response frequencies is not preserved in the older population is not conflicting with the finding of decreasing response amplitudes, both measures to a certain degree different descriptions of the same phenomenon. Interestingly, the 40-Hz response did not show any indication of decreasing phase synchrony with increasing age.

### Long-latency responses

Most studies of the P1–N1–P2 waves of the auditory evoked responses to gap stimuli typically use stimuli with marker durations that are much longer than those used in our study [42]–[44]. The reason for using stimuli with very long leading markers is to separate the response to a short gap from the onset response to the sound in time. However, detecting a gap in continuous sound is a very different task than detecting a gap after a brief leading marker as in our study. Leading markers of 10 and 40 ms duration had been used by Gage et al. [45] who reported a decrease in N1_m_ amplitude with increasing gap duration for the 10-ms leading marker stimulus. Modifications in the long latency response around 200 ms and a mismatch negativity response for infrequent presentation of gap stimuli within a sequence of more frequent no-gap stimuli has been reported when using stimuli similar to those used in our study [46].

P1_m_ amplitude and latency increased with age, which is consistent with results of our previous studies [47] specifically with P1_m_ changes observed with stimuli with longer inter-stimulus interval in the same population as in this study [22]. We found a tendency for increasing P1_m_ responses to gaps of larger duration and the P1_m_ specific to the onset of the trailing marker seems to increase with increasing age, like the onset P1_m_, however, those findings did not reach significance. The effects on P1_m_ were small because of the short time interval between onset of the leading and lagging marker. Other studies using a leading marker of several hundred of milliseconds duration showed significant effects of gap duration on the P1_m_ to the onset of the trailing marker, suggesting that the P1_m_ could be a sensitive indicator for the salience of the gap stimulus [42].

The interpretation of modifications of the P1_m_–N1_m_–P2_m_ complex by the gap is problematic because waves elicited by different stimulus features may overlap and cause an apparent change in peak amplitudes. By calculating difference waves between the gap responses and responses to no-gap stimuli, we were able to isolate a response waveform specific to the gap. In case of the long leading marker and long duration stimulus (40-16-20 ms) the difference wave showed characteristic P1_m_-N1_m_-P2_m_ deflections with similar patterns across age groups. This was a surprising result given that the N1_m_-P2_m_ waves to onset response was strongly reduced because of the fast repetition rate of stimuli. An explanation could be that the 40-16-20 ms stimulus was rather different from the other stimuli and occurred eight times less frequently than the onset response. The other gap stimuli may have been less different from each other and the no-gap stimuli and although all stimuli occurred with same probability, the effect of novelty may have been less expressed. Nonetheless at least in the younger group all types of gap stimuli elicited a difference wave with most prominent peak around 200 ms.

A main effect of aging was reflected in the difference waves, with the activity around 200 ms and later decreasing with advancing age. For the easiest gap condition with long duration leading marker and long gap it seems that the P2_gap_ response is reduced with increasing age. For the other stimulus conditions the waves were not easily identified. Furthermore, multiple components may contribute to the net effect on the difference waves. Even without addressing the amplitude differences to a certain component of the evoked response most interesting is the latency interval in which the age effects occurred. The latency interval following the N1_m_ response is the time in which an auditory object is recognized as indicated with the object related negativity [48]. The auditory input is compared to an internal representation and possible mismatch is signaled with the mismatch negativity [49], and when a task has to be performed in response to the auditory stimulus the analysis of the stimulus is expressed in the processing negativity during this time interval [50]. Thus, in contrast to mere registering the physical stimulus change, the activity around 200 ms the difference waves is most likely related to processing the gap as an integrated object.

We described with this study changes in brain activity related to variation of stimulus parameters in a gap detection experiment (i.e the durations of the gap and the leading marker) and how those changed with the age of healthy adults. We recorded the data in a passive listening experiment and thus did not provide behavioural measures for direct comparison of brain activity and individual performance. Recording gamma and high-gamma responses required a large number of experimental trials for a sufficient signal-to-noise ratio, which would not have been feasible with the long-duration trials of an active experiment within reasonable investigation time. Nevertheless, we demonstrated changes in the brain responses related to both the duration of the gap as well as the duration of the leading marker in all age groups. The effect of both parameters on gap detection performance is well documented in the literature. For the effects of aging, most importantly we showed that aging affected the brain responses at different levels. How those changes relate to behavioural changes needs further research and likely cannot be answered with a simple function of brain-behaviour relation because the aging brain is characterized by a large variability in the threshold at which a reduction of resources leads to impairment (i.e. brain reserve) and large variability in the capability of compensating for deficits (i.e cognitive reserve) [51]. Our finding of aging related changes at central level indicate that such inter-subject variability has to be considered in future studies and the methods and results of this study may have laid the groundwork for continuing research.

### Conclusion

Our results indicate that normal aging affects central auditory processing at a higher level than previously thought. We studied multiple components of the auditory evoked responses and how they are affected by task difficulty and the ages of the listeners. We isolated the 40-Hz response to the onset of the lagging marker, which is the key physical feature for detecting a gap in sound, and demonstrated that this response did not decrease with age. We identified two response components, high-gamma oscillations and difference waves in the 200-ms latency range, which are related more to the gap as an auditory object rather than acoustical features such as sound onsets. Both components were noticeably reduced with increasing age and this effect was already evident in the middle-aged group. Thus, we conclude that the auditory system performs well even in older listeners in the registration of relevant acoustical changes with high temporal acuity. Age-related difficulties in gap detection more likely occur at a later stage of processing where the acoustical properties of the stimulus are bound together to form an auditory object.

## Methods

### Participants

Seventeen young (mean age 24 years; range 20–36 years; 12 female), twelve middle-aged (mean age 47 years; 38–54 years; 6 female), and thirteen older (mean age 71 years; 61–81 years; 7 female) healthy adults, recruited from the local community and laboratory personnel, participated in the study. Although, all participants had pure-tone thresholds less than or equal to 30 dB hearing level (HL) between 250 and 2000 Hz in both ears, mean audiometric thresholds across this frequency range increased gradually between age groups (F(2,40) = 14.1, p<0.001) from 2.4 dB HL in the young adults, 4.5 dB HL in the middle-aged group, to 13.3 dB HL in the group of older adults, which was significantly higher compared to young or middle-aged adults (p<0.001 in both cases). Group mean audiometric thresholds are reported in Table 2. Participants provided written informed consent in accordance with the guidelines established by the University of Toronto and Baycrest Centre. The study had been approved by the Research Ethics Board at Baycrest Centre.

**Table 2:** Group mean audiometric (HL) threshold (and standard error) in young, middle-aged, and older adults averaged over the left and right ears.

	Frequency			
Groups:	250 Hz	500 Hz	1000 Hz	2000 Hz
Young	1.0 (1.9)	5.0 (1.5)	1.9 (1.3)	1.7 (1.7)
Middle-aged	1.9 (2.4)	7.7 (1.8)	4.4 (1.6)	4.2 (2.0)
Older	11.0 (2.3)	14.6 (1.7)	13.7 (1.6)	13.8 (1.9)

### Stimuli and Procedure

During three blocks of 13 min recording time each, participants listened passively to equal numbers of sound stimuli whose durations were 34, 46, 64, and 76 ms, with half of the stimuli containing a gap (Figure 1). Stimuli were 1-kHz pure tones with the envelope shaped by Gaussian pips with standard deviation of 0.5 ms as described by Schneider and Hamstra [14] and amplitudes adjusted for equal energy. Diotic stimuli were presented at 80 dB sound pressure level via an OB 822 Clinical Audiometer through ER30 transducers (Etymotic Research, Elk Grove, USA) and connected with 1.5 m of length matched plastic tubing and foam earplugs to the participant's ears. Sound transmission through plastic tubing of such length was required because the sound transducers had to be placed at a sufficient distance from the MEG sensor to avoid any interference between the stimulus signal and the recorded brain activity. Below 2000 Hz the frequency characteristic of the sound transmission system was relatively flat (±6 dB) and the phase characteristic was linear as tested using a 2-cc coupler. Sound transmission through the plastic tubes caused a delay of 10 ms. During the data analysis all estimated latencies were corrected for such delay.

The different types of stimulus sounds were presented in random order with the inter-stimulus interval uniformly randomized between 120 and 320 ms, equivalent to stimulus onset asynchrony (SOA) between 154 and 396 ms, under control of Neuroscan STIM Software. The mean SOA of 275 ms is short compared to the refractory time of N1_m_ -P2_m_ waves of the auditory evoked response. In addition, one block of 24 min recording time was presented, with longer SOA of 850 to 1850 ms, which allowed recovery of the N1_m_ -P2_m_ waves. The effect of stimulus duration on the long latency responses and how it changes with aging has already been reported [22]. No specific task was required for the subjects who watched a subtitled silent movie of their own choice.

### Data Acquisition and analysis

The MEG was recorded in a magnetically shielded room using a 151-channel whole head neuro-magnetometer (OMEGA, VSM Medtech Inc., Vancouver, Canada). In order to minimize head movement, participants were in supine position with their head resting in the helmet shaped MEG sensor. Neuromagnetic activity was sampled at a rate of 1250 Hz after 320 Hz low-pass filtering and was recorded continuously. We modeled the averaged MEG data with single dipoles in left and right auditory cortices and calculated the waveforms of cortical source strength [52]. All data analyses reported here were based on the bilateral source waveforms. Studying the sequence of positive and negative waves in the evoked responses is very much like the more conventional analysis of waveforms obtained from selected electrodes in EEG or sensors in MEG, and allows comparisons with earlier studies.

### Dipole source analysis

The MEG data were parsed into epochs including 200 ms of pre- and 600 ms of post-stimulus activity. Hence, the beginning and ending of each epoch may have overlapped with the previous or next epoch; however, a shorter interval around the stimulus onset was used for further analysis. A principal component analysis was performed on each epoch and components larger than 2.0 pT at any time point were subtracted from the data. This step of pre-processing effectively removed large artifacts caused by eye-blinks [53], [54]. Individual dipole source analysis was performed on the grand averaged data across all stimulus types after band-pass filtering between 18 and 80 Hz. The source model consisted of single equivalent-current dipoles in the left and right temporal lobes and was approximated to the 15 to 60 ms latency interval around the peak of gamma-band response to the stimulus onset. The head model for dipole fitting was a sphere with homogenous volume conduction, which was approximated to the individual head shape with best fit to temporal regions. Single dipoles in both hemispheres were fit simultaneously to the 151-channel magnetic field distribution. First, we modeled the data with a mirror symmetric pair of dipoles. The resulting source coordinates were then used as starting points to fit the dipole in one hemisphere while the coordinates in the other hemisphere remained fixed. We then switched between hemispheres and repeated the last step until the source coordinates showed no further change. Dipole fits were accepted if their calculated fields explained at least 90% of the variance of the measured magnetic field. Up to three estimates of auditory cortex source locations were obtained for each participant from three separate blocks of MEG recording. The median of spatial coordinates and orientations were used as individual models to measure the source waveforms for the auditory evoked responses. Source waveforms for each stimulus condition were calculated based on the fixed dipole locations and orientations. The polarity of source waveforms was defined according to the polarity of an AEP recorded from a vertex electrode in EEG such that the neuromagnetic counterpart of the N1 response showed a negative going wave. Similar dipole analysis was performed for the 24-Hz low-pass filtered magnetic fields recorded with long SOA stimuli in a time interval of ±15 ms around the peak of the N1_m_ wave. The resulting estimates of the N1_m_ source location in each individual participant were used for comparison with the source location for the gamma-band response only.

### Analysis of source waveforms

A time-frequency analysis based on a Morlet wavelet [55] was performed on the averaged source waveforms for identifying the various components of evoked activity. Three different filters, a 24-Hz low-pass, a 18-80-Hz band-pass, and a 72-98-Hz band-pass, were used to separate the low frequency, the gamma-band, and higher gamma-band responses of the source waveforms, respectively. All filters were fourth order Butterworth type recursive filters with zero time delay (using the MATLAB filtfilt function).

The durations of the P1_m_ -N1_m_ -P2_m_ responses or the gamma band responses were longer than the durations of markers and gaps in the stimuli. Thus, responses elicited by the stimulus onsets and offsets and responses related to a gap may partially overlap. Under the assumption that onset responses would be identical for the gap and no-gap stimuli, we subtracted the no-gap response from the gap response, which resulted in difference waveforms representing the effect of the gap. Difference waveforms were calculated for the 24-Hz low-pass filtered responses showing the P1_m_–N1_m_–P2_m_ response and the 18–80 Hz band-pass filtered waveforms showing the transient gamma band response. The higher gamma band responses were of short duration and did not overlap. Thus no difference waves were calculated for the high-gamma responses.

Group mean peak amplitudes and latencies and the 95% confidence intervals for the group means were estimated using a bootstrap procedure. Given *n* individual source waveforms, bootstrap samples were constructed by random selection of *n* waveforms with replacement. The samples were averaged and the first derivative was calculated. The time points of zero crossing in the first derivative going from positive to negative were identified as latencies of a maximum in the waveform and time points of negative to positive zero crossings indicated minima in the waveforms. This procedure was repeated 1000 times, and the 50%, 2.5%, and 97.5% values of the distributions for peak amplitude and latencies were accepted as estimates for the group means, and their 95% confidence limits, respectively.
